# Humidity modifies species‐specific and age‐dependent heat stress effects in an insect host‐parasitoid interaction

**DOI:** 10.1002/ece3.70047

**Published:** 2024-07-21

**Authors:** Dongbo Li, Benjamin Brough, Jasper W. Rees, Christophe F. D. Coste, Chenggui Yuan, Mike S. Fowler, Steven M. Sait

**Affiliations:** ^1^ School of Biology, Faculty of Biological Sciences University of Leeds Leeds UK; ^2^ Department of Biosciences Swansea University Swansea UK; ^3^ Department of Mathematics Swansea University Swansea UK

**Keywords:** bottom‐up effect, environmental stress, extinction, insect decline, phenotypic traits, species interactions

## Abstract

Climate change is projected to increase the frequency and intensity of extreme heat events, and may increase humidity levels, leading to coupled thermal and hydric stress. However, how humidity modulates the impacts of heat stress on species and their interactions is currently unknown. Using an insect host‐parasitoid interaction: the Indian meal moth, *Plodia interpunctella*, and its endoparasitoid wasp, *Venturia canescens*, we investigated how humidity interacted with heat stress duration, applied at different host developmental stages, to affect life history traits. Hosts parasitized as 4th instar larvae and unparasitized hosts were maintained in high‐ (60.8% RH) or low‐humidity (32.5% RH) at constant 28°C. They were then exposed to a 38°C thermal stress with a duration of 0 (no heat stress), 6 or 72 h in either the 4th or 5th host instar. Neither humidity nor heat stress duration affected emergence of unparasitized hosts, but increasing heat stress duration during the 4th instar decreased parasitoid emergence irrespective of humidity. When applied during the 5th instar, increasing heat duration decreased parasitoid emergence under low humidity, but no effect of heat stress was found under high humidity. Moreover, experiencing longer heat stress in the 4th instar increased host larval development time and decreased body size under high humidity, but this effect differed under low humidity; increasing heat duration in the 5th instar decreased parasitoid body sizes only under low humidity. Larval stage and heat stress duration directly affected parasitized host survival time, with a concomitant indirect reduction of parasitoid sizes. We show that humidity modifies key life history responses of hosts and parasitoids to heat stress in species‐specific ways, highlighting the potential importance of humidity in regulating host‐parasitoid interactions and their population dynamics. Finally, we emphasize that interactions between environmental stressors need to be considered in climate change research.

## Graphical abstract

Understanding how species and their interactions respond to extreme heat events is important to evaluate the impact of climate change. Using a well‐established host‐parasitoid system, we show that the life history responses of hosts and parasitoids to heat waves can largely depend on humidity levels.

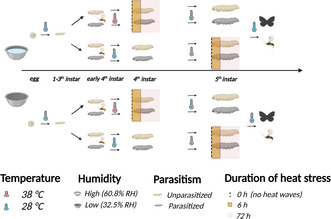

## 1 INTRODUCTION

Climate warming, manifested primarily through increasing temperature and the greater frequency and intensity of heat stress events, such as short‐lived extremes or heat waves (Rahmstorf & Coumou, 2011; Sun et al., 2019), has widespread impacts across ecological scales. How extreme heat waves affect biodiversity and ecosystems has yielded mixed findings (Cope et al., 2023; Li et al., 2017; Ruthrof et al., 2018) as extreme high temperatures have been shown to have positive, negative, or neutral effects on the individual fitness of living organisms, such as insects (Ma et al., 2021; Skendžić et al., 2021). However, as insects' responses to thermal stress may not be consistent throughout ontogeny, the timing and duration of heat stress events experienced at different life stages may lead to contrasting fitness outcomes (Moore et al., 2022; Valls et al., 2020). Furthermore, differences in thermal tolerance between interacting species may result in differential responses to high temperatures, with trophic interactions in particular likely being disrupted (Bannerman & Roitberg, 2014; Moore et al., 2021). Hence, investigating how extreme high temperature affects insects at different life stages and their trophic interactions is important to understand how communities respond to recent and future climate change.

In addition to extreme temperatures, climate warming may increase the rate of surface evaporation, resulting in a combined stress of high heat and high humidity (Schär, 2016). Such ‘humid heat waves’ have been observed in many parts of the world and are projected to escalate under future climate change scenarios (Gershunov & Guirguis, 2012; Russo et al., 2017; Wang et al., 2021). However, despite its pronounced influence on regulating insect life cycles, evidence for how humidity modifies thermal responses is underdeveloped (Brown et al., 2023), leading to uncertainty about their combined effects on the abundance and distribution of species (Chown et al., 2011; Simmons et al., 2023). Within this context, efforts to forecast the persistence of trophic interactions and their dynamics may be limited (Brown et al., 2023; Rozen‐Rechels et al., 2019).

Humidity levels determine the availability of water vapour that insects acquire from the environment, and they affect the ability to regulate water loss through spiracular respiration (Shipp et al., 1988). For example, when exposed to low humidity environments, adult cockroaches (*Naupheota cinera*) exhibited a slower rate of water loss due to longer periods of spiracular closure (Schimpf et al., 2009). However, some insects gain moisture when air humidity is increased, subsequently lowering the risk of desiccation (e.g. Johnson, 2010; Salin et al., 1999). Given that temperature drives many physiological processes, including spiracular control, the responses of insects to humidity may also be temperature‐dependent (Heinrich & Bradley, 2014). If the control of spiracles is an adaptative mechanism to balance the trade‐off between water loss and gas exchange in changing environments (Oladipupo et al., 2022), humidity will likely affect insect survival when temperatures increase.

Parasitoids are top‐down regulators of their host's population dynamics, they are a key component of terrestrial food webs and important in biological control (Furlong & Zalucki, 2017; Jeffs & Lewis, 2013). Koinobiont endoparasitoids lay their eggs inside their host, and their larvae continue to develop and feed inside the host as it grows and reaches the optimal size for adult parasitoid eclosion. There is a growing body of evidence that temperature can have both a direct impact on koinobiont endoparasitoid life history, and an indirect impact via the response of their hosts to temperature (e.g. Abarca & Spahn, 2021; Cavigliasso et al., 2021; Meisner et al., 2014). In general, parasitoids are less tolerant of high temperatures than their hosts (Abarca & Spahn, 2021; Furlong & Zalucki, 2017), and hosts may suppress parasitism if parasitoids experience extreme high temperatures during early development stages (Moore et al., 2022). On the other hand, endoparasitoids may adapt to sublethal temperatures, via multiple ontogenetic responses determined by host development (Harvey et al., 1994; Harvey & Strand, 2002). Additionally, as hosts are the only source of moisture for endoparasitoid larvae, humidity may indirectly affect parasitoids through the direct impact of humidity on their host. Previous work has shown that interactions between environmental factors, such as fluctuating temperatures and resource quality, influence the life history traits of both hosts and parasitoids in ways that could not be predicted from each factor alone (Mugabo et al., 2019), emphasizing why it is important to consider combinations of environmental factors and their impacts on interacting species.

**FIGURE 1:**
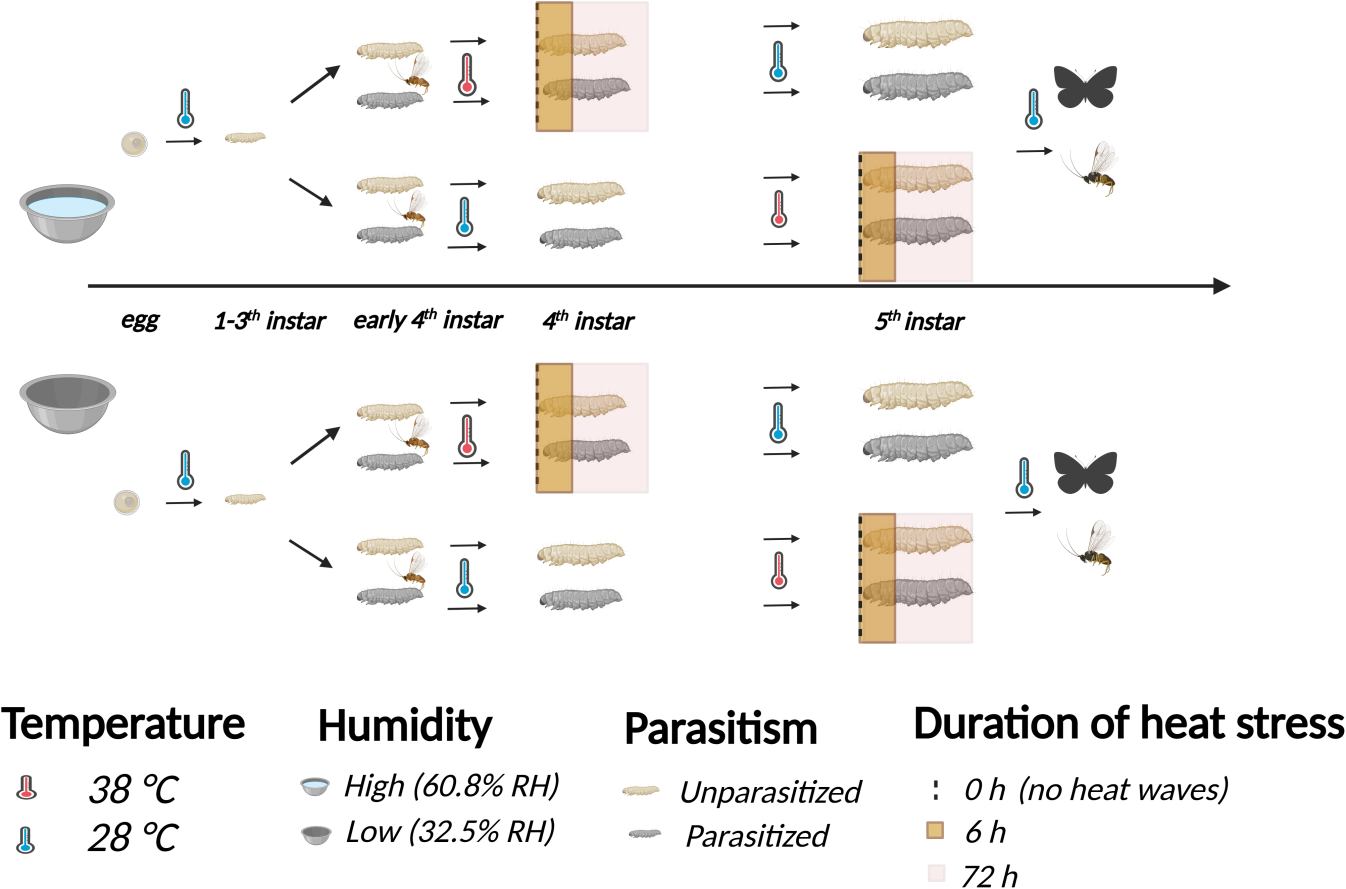
Schematic diagram of the experimental design. Host eggs were placed in either a high‐humid or low‐humid incubator at a constant temperature of 28°C until they reached the early 4th instar. Half of the hosts were parasitized, with the other half left unparasitized. Both parasitized and unparasitized hosts experienced a 38°C heat stress, for 0 (no heat stress control), 6 or 72 h, either as early 4th instar or 5th instar larvae. After exposure to the heat stress, all the hosts were kept at constant 28°C until adult emergence.

We investigated the effect of humidity on the responses of an insect host and its parasitoid when they were exposed to heat stress of different durations and experienced at different host ages, using the Indian meal moth *Plodia interpunctella* and its koinobiont endoparasitoid wasp *Venturia canescens*. We carried out a single‐generation life‐history experiment where hosts were kept individually either in a high or low humid environment at a constant temperature of 28°C, with or without being parasitized early in the fourth instar. All parasitized and unparasitized hosts subsequently experienced either no heat stress (0‐h control), a 6‐, or 72‐h heat stress of 38°C, applied either in the host fourth or fifth instar. We measured key life history traits of both hosts and parasitoids (larval development time, adult emergence and body size) to evaluate how these traits responded to heat stress and humidity individually, and then assessed whether these responses were modified by a high humid environment (see Figure 1 for experimental design).

We examined the direct effects of heat stress on hosts and parasitoids, and the indirect effects through their trophic interaction, to investigate if the direct and indirect pathways of heat stress on species were different between humidity levels. We predicted that (1) increasing heat stress duration would negatively affect hosts and parasitoids; (2) parasitoids will be more affected by heat stress than their hosts when they are in an earlier stage of their development; (3) humidity will modify heat stress effects on the hosts directly, and modify heat stress effects on parasitoids indirectly.

## 2 MATERIALS AND METHODS

### 2.1 Study organisms

The laboratory culture of the host *P. interpunctella* and larval hosts parasitized by *V. canescens* larvae were kept in temperature‐controlled incubators at University of Leeds, UK, under a constant temperature of 28°C and 16:8 light:dark cycles (Jones et al., 2015; Mugabo et al., 2019). The *P. interpunctella* colony has been reared under identical laboratory conditions for over two decades. Adult *V. canescens* were kept at ambient temperature, with a sugar water solution for maintenance. Larval hosts were provided with a wheat bran diet (as same with Mugabo et al., 2019). No artificial source of moisture was provided inside the incubator for the laboratory culture (Figure S1).

### 2.2 Experimental procedure

We conducted a life history experiment where hosts were kept individually for their entire life cycles. Eggs were collected for 24 h from approximately 50 randomly selected newly emerged host adults from the laboratory culture (Jones et al., 2015). The eggs were then transferred into 25‐well (5 cm × 5 cm) clear plastic plates (Sterilin Limited, Thermo Fisher Scientific, UK), with a single egg plus 0.3 g food added into each cell, which is sufficient for complete host development (Mugabo et al., 2019). A piece of 2‐ply tissue paper and a nylon mesh were placed between the wells and lid to provide ventilation and to prevent host larvae from moving between cells or escaping.

A total of 1360 eggs were assigned to the different treatment combinations. Half of the eggs (i.e. 680 eggs) were placed in an incubator with artificially increased humidity (i.e. high humidity), and the other half placed in an incubator with no humidity manipulation (i.e. low humidity), both at a constant 28°C with 16:8 light:dark cycle. These eggs were randomized beforehand and placed in the experimental incubators with and without increased humidity on the same date. Humidity was increased by placing ~1000 mL of a saturated sodium chloride solution in the humidity treatment incubators (Solomon, 1951). Temperature and humidity levels in the incubators were monitored using EasyLog‐USB‐2 data loggers (LASCAR electronics). Pilot data showed that at a constant temperature of 28 or 38°C the NaCl solution increased the relative air humidity (RH) from 30 ± 5% in the incubators with no solution to 60 ± 5% with the solution (Figure S1).

Larvae were checked daily to identify when they were at either the 4th or 5th instar. To avoid changes in the microclimate during monitoring of host development inside the 25‐well plates, which may affect their development rates, 200 extra eggs were set up following the same protocol and monitored to estimate the overall developmental status of the experimental cohort. When host larvae had reached their early fourth instar, half of them were parasitized in each humidity treatment (i.e. 340 eggs per humidity treatment; *hereafter* ‘H‐P’ treatment) and half were unparasitized in each humidity treatment (*hereafter* ‘H’ treatment). To parasitize hosts in the ‘H‐P’ treatment, we took each host out of its cell, and placed it in a petri dish under normal laboratory conditions. A newly emerged adult parasitoid wasp from the stock culture was placed with a host until it was parasitized, which was confirmed when *V. canescens* performed a characteristic cocking behaviour (Rogers, 1972). Each parasitized host was put back into its original cell and original treatment to continue development.

170 parasitized hosts and 170 unparasitized hosts in a high‐humid or low‐humid environment were then exposed to a 38°C heat stress in their fourth instar (immediately after parasitizing), while the rest were kept in their original incubators until they were exposed to the heat stress in their fifth instar (~5th day after parasitizing). Parasitized and unparasitized larvae were placed into incubators at a constant 38°C incubators (either with or without artificially increased humidity) for durations of 6 or 72 h. This was to simulate a real‐world scenario in the UK where species may experience hot weather events in a half‐day or in a continuous heat wave. Larvae were returned to 28°C and corresponding humidity conditions, and monitored daily until host or parasitoid adults emerged and died naturally. Control parasitized and unparasitized larvae were kept at 28°C (i.e. no heat stress) with high or low humidity. Collectively, the treatments comprised unparasitized hosts and hosts parasitized in the 4th instar, which were kept in a high‐humid or low‐humid environment, and exposed to a 38°C heat stress of durations of 0, 6, or 72 h in their fourth or fifth instar. There were 50 individual larvae for the 0‐h treatment, 60 larvae for the 6‐h heat stress, and 60 larvae for the 72‐h heat stress, in each combination of humidity × parasitism × larval stage.

We measured the following traits in unparasitized hosts: (1) host emergence, (2) juvenile development time (i.e. from egg to adult emergence), and (3) mid‐femur length (which is correlated with body size, Jones et al., 2015; Mugabo et al., 2019). For parasitized hosts, we measured (1) relative emergence success of hosts (i.e. parasitoids were killed by heat stress; where natural encapsulation by hosts was not considered), wasps (i.e. parasitoids survived and killed the host), or neither (i.e., both were killed); (2) wasp juvenile development time (i.e., from parasitism to wasp emergence); and (3) hind tibia length (as a measure of adult body size, Harvey et al., 2001). Legs were removed under a dissection microscope and measured to the nearest 0.001 mm using a Nikon DS‐5 M camera and the NIS‐element Dv2.3 software (Nikon instruments).

### 2.3 Data analysis

Data consisted of multiple trait measurements of unparasitized and parasitized hosts as responses, with humidity level, larval stage, and duration of heat stress as predictors. Humidity and larval stage were categorical variables, each with two levels, and duration of heat stress was treated as a continuous variable on a log_*e*_(x + 1)‐transformed scale. The number of wasps that emerged from hosts parasitized as 5th instar larvae without heat stress (i.e., control group for H‐P) were less than the minimum requirements for statistical tests (i.e. <3). Therefore, we increased the number of observations in this group by shuffling all no‐heat stress controls within each humidity treatment and split into two larval stages with equal numbers, using ‘sample_n()’ function in the ‘tidyverse’ package (Wickham et al., 2019). Statistical analyses were conducted using R (v 4.3.2, RCORETEAM, 2022).

We explored how humidity level modified the effect of larval stage and heat stress duration on adult emergence of unparasitized hosts using a generalized linear model (GLM) with a binomial error distribution. For parasitized hosts, we used a multinomial logit regression model to investigate if the relative success of hosts and parasitoids was affected by humidity level, larval stage under stress, duration of heat stress, and their interactions, using the ‘nnet’ package (Ripley et al., 2016). Success was measured as the three possible outcomes of each parasitized host (i.e., parasitoid emergence, host adult emergence, or no emergence). To compare how the survival of parasitoids differed between treatments, we used the outcome of ‘parasitoid emergence’ as the reference group. The effect of experimental treatments on the multinomial probabilities was assessed using the Anova() function with type III sums of squares from the ‘car’ package (Fox & Weisberg, 2019). The marginal trends (slopes) on the linear scale of heat stress duration interacting with larval stage and humidity levels were estimated using ‘emtrends()’ function in the ‘emmeans’ package (Lenth, 2024), followed by pairwise post‐hoc comparisons (with the ‘pairs()’ function) to assess whether these trends differed between experiencing heat stress as host 4th and 5th instar, and between high and low humidity.

We then examined how treatments affected the life history traits of surviving hosts and parasitoids. Host and parasitoid larval development were assumed to be gamma‐distributed under constant temperature (Li et al., 2022); therefore, GLMs were fitted with a log‐link Gamma error distribution for hosts and parasitoids that emerged as adults. Body size of unparasitized adult hosts and parasitoids were analyzed using GLMs with a log‐link gaussian error distribution. Humidity, larval stage, duration of heat stress, and their interactions were included as independent variables in these models. Model residuals were checked using a simulation‐based approach by running *n* = 250 simulations in ‘DHARMa’ package (Hartig & Hartig, 2017) and tested for normality using Kolmogorov–Smirnov test.

Finally, we investigated the direct and indirect effects of humidity, larval stage, and duration of heat stress on hosts, and how they subsequently affected the phenotype of parasitoids (body size) using a piecewise structural equational model (‘piecewiseSEM’ package, Lefcheck, 2016). Piecewise SEMs can switch from global to local estimation, which allows the fitting of equations with a range of distributions (Shipley, 2000). As the emergence of parasitoids resulted in the death of the host, we quantified the host's contribution to parasitoid size using host larval survival time (i.e. the date of parasitoid emergence minus the date of host egg laying). The hind tibia length of parasitoids was used as a measure of parasitoid performance to avoid the correlation between two endogenous variables (i.e. host survival time and parasitoid size). As such, our piecewise SEM included two underlying equations which specified (a) the effect of larval stage and duration of heat stress on host survival time and (b) the effect of host larval survival time, duration of heat stress, and host larval stage on the hind tibia of parasitoids. Both equations were fitted by a GLM with a log‐link gaussian distribution (Gamma GLMs were not supported by this R package), with model assumptions checked by the ‘DHARMa’ package (Hartig & Hartig, 2017). To examine if the effect of heat stress on hosts and parasitoids were modified by humidity, we performed a multigroup analysis incorporating humidity as a grouping variable. While constraining both paths may result in a saturated model (i.e. having 0 degrees of freedom to evaluate model fit), the process of automatically adjusting coefficients in piecewise SEMs allows us to examine if any of the paths varied among groups (Lefcheck, 2016). Thus, any significant interactions indicated whether heat waves interacted with humidity, and any constrained effects implied the path to the hosts or parasitoids did not differ with humidity. Standardized coefficients were calculated for non‐categorical variables.

## 3 RESULTS

**FIGURE 2:**
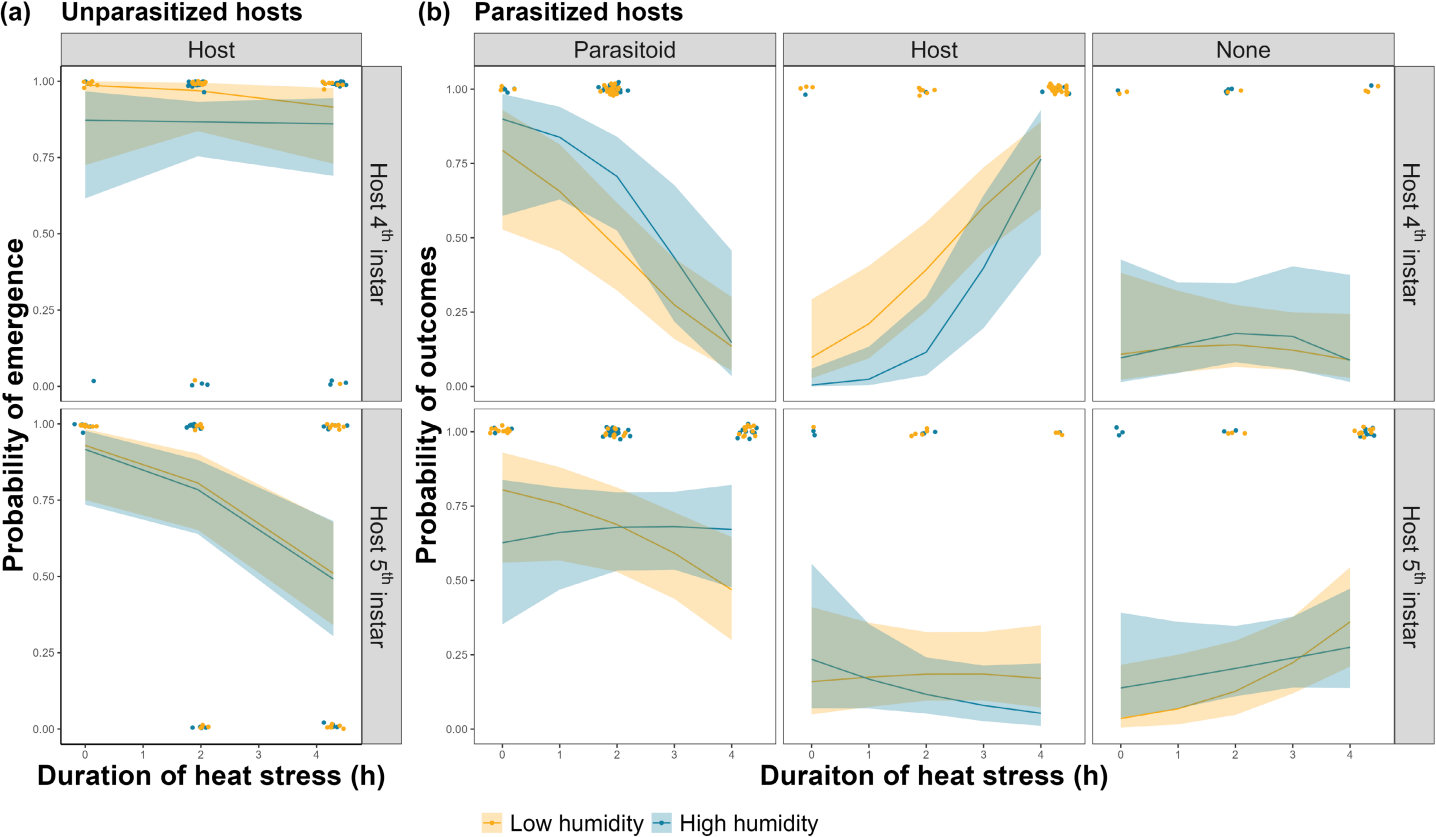
(a) Binomial GLM predicted values for the probability of adult emergence in unparasitized hosts as a function of heat stress duration applied during the host 4th (top panel) or 5th instar (bottom panel), under high‐ (blue) or low‐humidity (yellow) conditions. Jittered data points show whether host adults have emerged (i.e. 0 or 1) in each treatment combination. (b) Multinomial logit GLM predicted values for the probabilities of relative adult emergence success as a function of heat stress duration applied during the host 4th (top panels) or 5th instar (bottom panels), under high‐ (blue) and low‐humidity (yellow) for the parasitoid (left two panels), the host (middle two panels), or neither species (right two panels; i.e. complete mortality). Jittered data points show the number of each outcome (i.e. parasitoid, host, or none) in each treatment combination. Duration of heat stress (hours) is on a log_*e*_(x + 1) transformed scale. Probabilities were predicted by the full 3‐way interaction model, with 95% confidence limits shown as shaded intervals.

There was no effect of duration of heat stress, larval stage, or humidity level, nor their two‐ and three‐way interactions, on adult emergence in unparasitized hosts (binomial GLM, all *p* > .05, Tables S1–S3; Figure 2a). However, in parasitized hosts, the emergence success of two insects was significantly affected by experimental treatments in species‐specific and age‐dependent ways (Table 1; Table S4; Figure 2b). While humidity had no effect, duration of heat stress interacted significantly with larval stage (Table 1), with increased heat stress duration leading to reduced parasitoid emergence, and a corresponding increase in host emergence, when applied in the 4th instar under both humidity levels (Tables 1 and 2; Figure 2b). Notably, 72 h of heat stress applied in the 4th instar resulted in complete parasitoid mortality irrespective of humidity. In contrast, when heat stress was applied in the 5th instar, increased duration decreased parasitoid emergence and increased instances where there was no emergence of adult moths or wasps under low humidity, but this was not found with high humidity (Table 2). In addition, experiencing heat stress during the 5th instar did not affect host emergence irrespective of humidity levels. Post hoc comparisons of emergence showed significant differences in slopes between 4th and 5th host instars under both humidity levels (4th instar – 5th instar, pairwise comparison within hosts, both *p* < .05, Table S5). The difference in slopes of parasitoid emergence between hosts parasitized as 4th and 5th instars was only found with high humidity (4th instar – 5th instar, pairwise comparison within parasitoids, *p* < .05, Table S5; more in Table S6).

**TABLE 1:** Anova (type III sums of squares) table of the multinomial logit regression model.

Predictors	*χ* ^2^	df	*p*
Humidity	5.2497	2	.072
Duration	19.9966	2	**<.001**
Larval stage	1.2054	2	.547
Humidity: duration	2.6381	2	.267
Humidity: larval stage	5.1476	2	.076
Duration: larval stage	8.4971	2	**.014**
Humidity: duration: larval stage	4.3116	2	.116

*Note*: Significant effects were in bold.

**TABLE 2:** The estimated marginal linear trends (and 95% confidence limits; Lenth, 2024) of heat stress duration applied to different larval stages under low‐ and high‐humidity levels, on the change in probability of three possible emergence outcomes in parasitized hosts: parasitoid wasp emerges, host moth emerges, or neither emerges (‘none’; i.e. mortality of both).

Outcomes	Humidity	Larval stage	Trend	SE	df	Lower CL	Upper CL
Parasitoid	Low	4th instar	***−0.452***	0.167	16	*−0.806*	*−0.098*
Host	Low	4th instar	**0.510**	0.159	16	0.174	0.846
None	Low	4th instar	*−0.058*	0.197	16	*−0.476*	0.360
Parasitoid	High	4th instar	***−0.720***	0.259	16	*−1.27*	*−0.171*
Host	High	4th instar	**1.01**	0.290	16	0.395	1.62
None	High	4th instar	*−0.289*	0.286	16	*−0.895*	0.316
Parasitoid	Low	5th instar	***−0.288***	0.131	16	*−0.566*	*−0.011*
Host	Low	5th instar	*−0.137*	0.161	16	*−0.479*	0.206
None	Low	5th instar	**0.425**	0.188	16	0.027	0.823
Parasitoid	High	5th instar	0.078	0.141	16	*−0.222*	0.377
Host	High	5th instar	*−0.310*	0.215	16	*−0.766*	0.146
None	High	5th instar	0.232	0.173	16	*−0.135*	0.600

*Note*: Trend <0: decrease in emergence probability for focal species with increasing heat stress duration (italics); trend >0: increase in emergence probability with increasing heat stress duration; trend estimates formatted in bold showed significant difference from 0 at *p* < .05.

**FIGURE 3:**
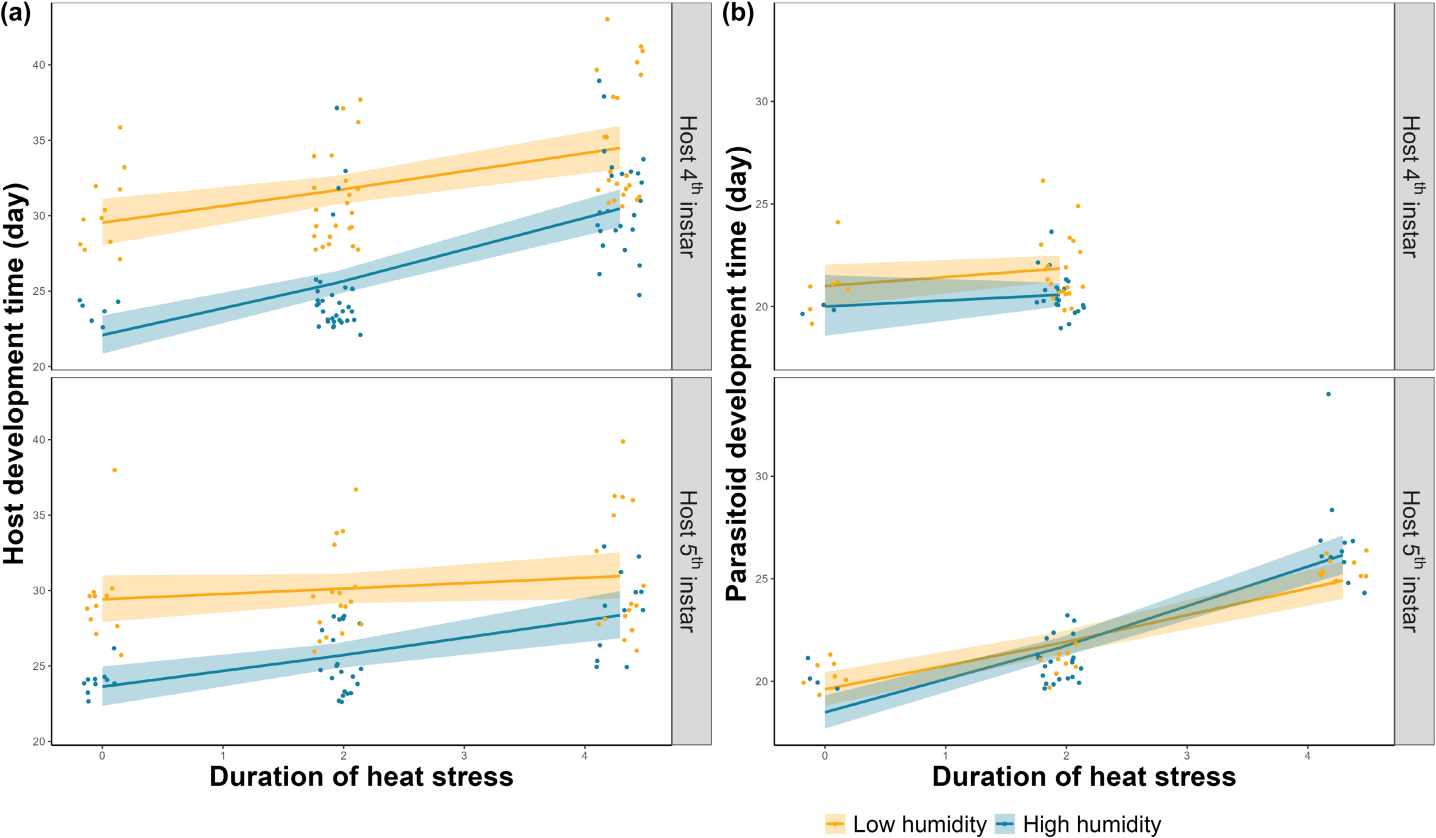
Larval development time (days) of (a) unparasitized hosts and (b) parasitoids as a function of heat stress duration. Hosts developed in either high‐humidity (blue) or low‐humidity (yellow), experiencing heat stress duration at either host 4th instar (top panels) or 5th instar (bottom panels). Data were jittered as points for clarity. Probabilities were predicted by the full 3‐way interaction model, with 95% confidence limits shown as shaded intervals. Note that in (b) parasitoid mortality was 100% when the heat stress was applied in 4th instar larvae for 72 h, so there are no data or model predictions for larval parasitoid development.

Larval development time of unparasitized hosts was significantly affected by heat stress duration, humidity, and their two‐way interaction (log‐link Gamma GLM, all *p* < .05, Tables S7 and S8). Unparasitized host larval development was overall faster under high humidity conditions when no heat stress was applied (Figure 3a), but slower when heat stress duration increased in the 4th instar. When heat stress was applied during the 5th instar at high humidity, there was no effect of heat stress duration on larval development time (Table S9). Moreover, when heat stress was applied during 4th instar, high humidity significantly increased the effect size (slope) of heat stress duration (Table S10; Figure 3a), suggesting that the effect of heat stress duration on 4th instar host was modified by humidity.

For parasitoids that successfully emerged, heat stress duration and larval stage interactively affected larval development time, but this was not moderated by humidity levels (log‐link Gamma GLM, *p* < .05, Tables S7 and S8). Increasing heat stress duration increased the larval development time of parasitoid under both humidity levels, when heat stress applied during 5th instar (Table S9; Figure 3b), but there was no effect when heat stress was applied during the 4th instar. Notably, at the high humidity level, the effect size (slope) of heat stress duration was smaller in the 5th than the 4th instar (Table S8; Figure 3b), suggesting a more stage‐specific effect of heat stress duration on the development of parasitoids.

**FIGURE 4:**
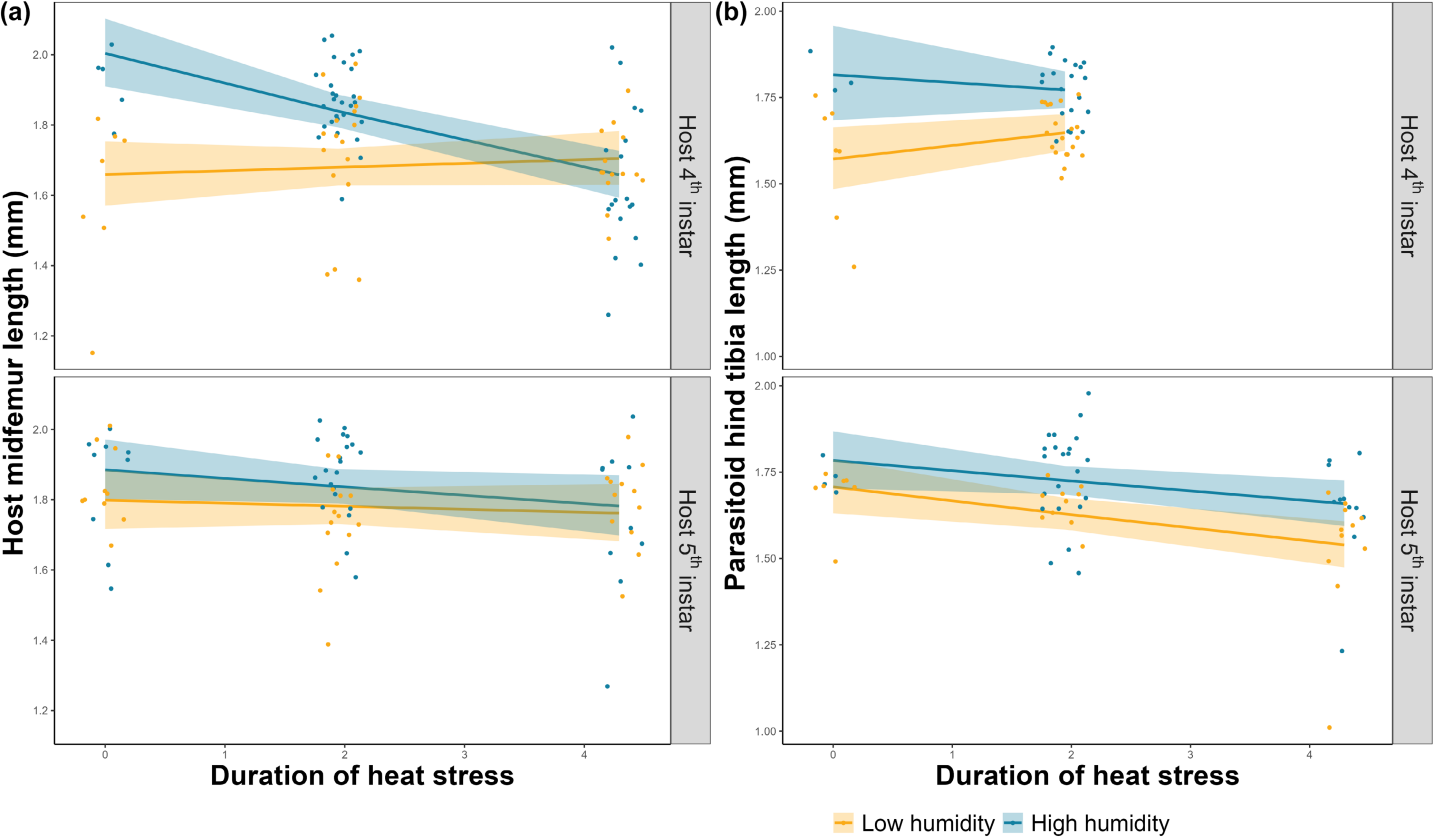
(a) Mid femur length of hosts and (b) hind tibia length of parasitoids as a function of heat stress duration. For annotations, see legend for Figure 3. Note that in (b) parasitoid mortality was 100% when the heat stress was applied in 4th instar larvae for 72 h so there are no data or model predictions for adult parasitoid hind tibia length.

Humidity, larval stage, and heat stress duration interactively affected the mid femur of unparasitized hosts (log‐link gaussian GLM, all *p* < .05, Tables S11 and S12; Figure 4a). Specifically, at high humidity, increasing heat stress duration during the 4th instar significantly decreased mid femur length, but no effects were found in other combinations (Table S13). The slope of heat stress duration when applied during the 4th instar was larger under high than low humidity (Table S14; Figure 4a). This means that when experiencing heat stress during the 4th instar, increasing duration had no effect on the body size of unparasitized hosts when humidity was low, but body size decreased when humidity was high.

The hind tibia length of parasitoids was significantly affected by humidity, and the interaction between larval stage and duration (log‐link gaussian GLM, all *p* < .05, Tables S11 and S12). Parasitoids were overall larger if the host developed in high humidity conditions, or experienced heat stress during the 5th rather than the 4th instar (Table S12; Figure 4b). However, increasing heat stress duration significantly decreased the hind tibia length of parasitoids if heat stress was applied during the 5th instar under low humidity conditions. No effects were found in other combinations (Table S13), nor effect sizes (slopes, Table S14).

**FIGURE 5:**
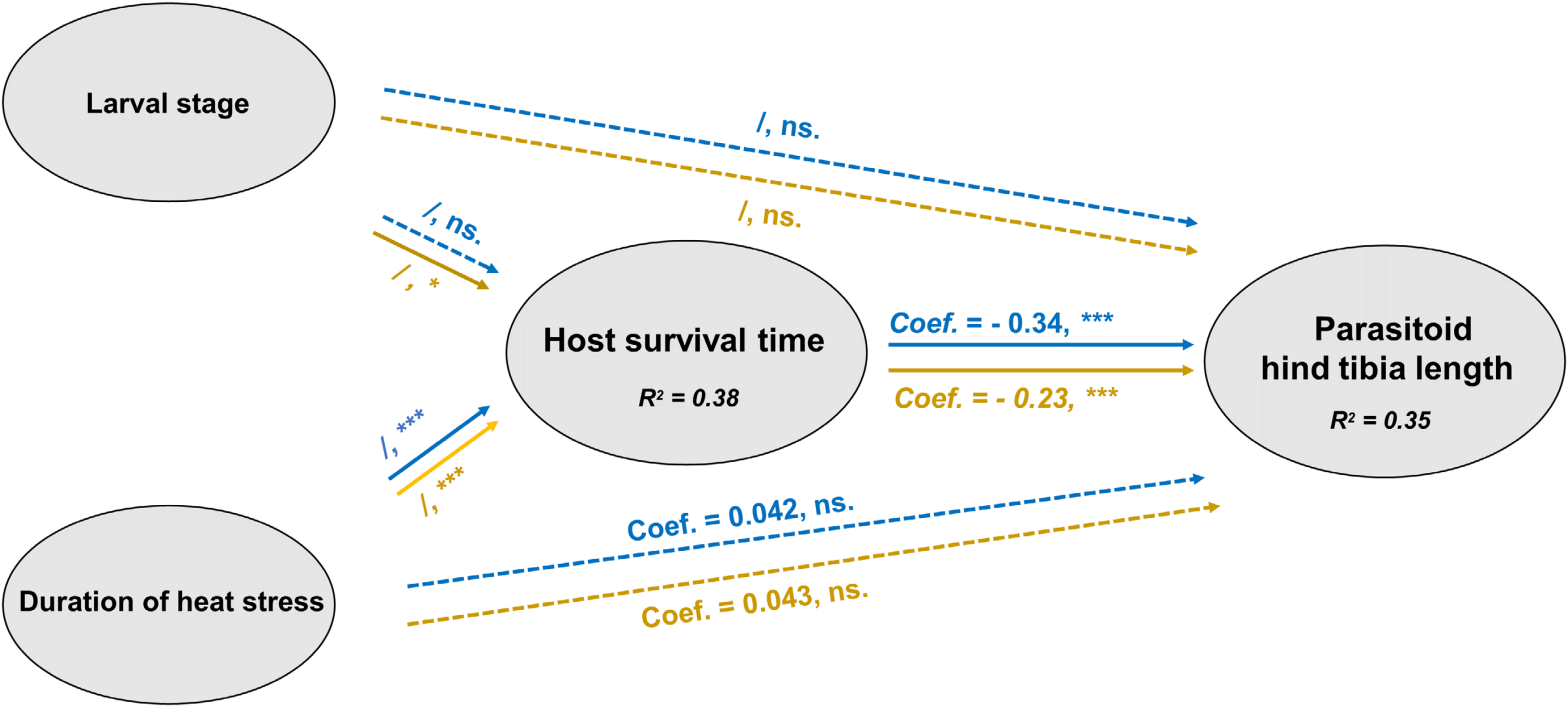
Multigroup structural equation model results showed both larval stage and duration of heat waves directly affected host survival time, and indirectly affected the size of parasitoids through the host response. The direct and indirect effect of larval stage and duration of heat waves on the *Plodia—Venturia* host‐parasitoid system depended on whether they developed in high humidity (blue) or in low humidity (yellow). Significant paths are denoted with solid arrows whereas non‐significant paths are denoted as dashed arrows. Standardized coefficients (/ when standard coefficients were unavailable) and significant levels are given on the top or bottom of the arrows.

The structural equation model showed that the timing (host larval instar) and duration of heat stress directly affected the survival time of hosts, but this did not have an indirect effect on the size of parasitoids (Figure 5). Instead, the hind tibia length of parasitoids was negatively correlated with host survival time (Figure 5), suggesting an indirect effect of larval stage and duration of heat stress on the size of parasitoids. All the paths to parasitoids were constrained to a global estimation (*p* > .05, more in Appendix Table S15), indicating that the effect of heat stress and host survival time on parasitoid hind tibia length did not depend on humidity. However, the unconstrained paths to hosts indicated that the effect of heat stress on host survival time depended on humidity levels (*p* < .05, Appendix Table S14 and S16). In low humidity conditions, host survival time was affected by larval stage and duration of heat stress, while it was only affected by duration of heat stress in high humidity conditions.

## 4 DISCUSSION

To our knowledge, empirical evidence for how humidity regulates the responses of interacting species to extreme temperatures is limited. We investigated how the life history traits of two insects, a host and its parasitoid were affected by different timing and duration of heat stress, and how these effects were modified by different humidity levels. We showed that humidity interacting with heat stress can strongly affect some aspects of the life histories of hosts and parasitoids, with a direct effect on hosts and an indirect effect on parasitoids.

### 4.1 Species‐specific and age‐dependent effects of heat stress duration

While duration of heat stress did not affect the probability of host emergence directly, exposing the parasitized hosts to longer heat stress in the early life stage increased the mortality of parasitoids, including complete mortality after 72 h of exposure to heat stress, resulting in greater host emergence from parasitized hosts. This supports the general finding that parasitoids are less tolerant to heat stress than their hosts (Hance et al., 2007; Wenda et al., 2023), but emphasizes that heat stress duration and timing in particular may be fundamental to parasitoid success. Thus, the detrimental effects on parasitoids experiencing high temperatures for a long period of time can disrupt trophic interactions, and suggests that pest outbreaks might be more likely if such conditions are experienced in nature (Moore et al., 2022). In addition, the larval development time of hosts and parasitoids that survived was generally longer when exposed to longer heat stress, indicating an increased energy expenditure for hosts and parasitoids in sublethal or short duration heat stress environments. Hosts and parasitoids may be able to cope with heat stress by accumulating heat shock proteins and biogenic amines (González‐Tokman et al., 2020), allowing them to acclimate to the new environment, but this is associated with an extra energetic cost. This cost may result in prolonged developmental time (Gillespie et al., 2012; Zhang et al., 2019) and reduced fitness in the adult stage (Nguyen et al., 2013; Yu et al., 2022) as life history trade‐offs.

The timing of heat stress is known to be important for host‐parasitoid interactions, as some development stages of parasitoids are more sensitive to heat stress than other stages (Simaz & Szűcs, 2021; Valls et al., 2020; Zhang et al., 2019). In accordance with our predictions, we showed that experiencing longer heat stress at an early stage (4th instar) increased parasitoid mortality, but had no effect on unparasitized hosts, suggesting that experiencing heat stress at an early stage of ontogeny may be critical to parasitoid survival. Moore et al. (2021) found that compared with other larval stages, experiencing heat waves at the embryonic stage resulted in complete mortality of the parasitoid *Cotesia congregata*. In our system, *V*. *canescens* starts to build body mass after *c*. 5 days following parasitism of *P. interpunctella* (Harvey et al., 1994), suggesting that the parasitoids may still be in the embryonic stage when they experienced heat waves during the 4th instar. Furthermore, the timing of heat stress could modify the life history of surviving parasitoids (Simaz & Szűcs, 2021; Zhang et al., 2019), as we also showed that parasitoids experiencing heat stress at a later stage had shorter larval development time and increased their hind tibia length. Although the physiological mechanisms underpinning these life history changes were not clear, our results highlighted that the effect of heat stress on the success of parasitism was stage specific.

### 4.2 Humidity modifies host‐parasitoid heat stress effects

Of potential critical importance for the maintenance of host‐parasitoid interactions was our finding that a high humidity level maintained parasitoid emergence when long heat stress duration was experienced, but only during the host 5th instar. In contrast, high humidity did not prevent significant declines in parasitoid emergence with increasing heat stress duration earlier in development. Furthermore, a faster development rate in the host under high humidity may result in a shorter generation time and narrower parasitism window, leading to more frequent and severe pest outbreaks. However, when heat stress was applied, the positive effect of humidity could not fully offset the negative effect of heat stress to the host. On the contrary, humidity could aggravate the negative effect of heat stress duration especially on early larval instars, as our results showed a higher increase in larval development time, and a significant reduction in mid femur length with increasing heat stress duration in the 4th instar in high rather than low humidity. In contrast, the effect of heat stress duration on parasitoids seems more stage‐specific, as trait modifications in parasitoids were likely found when heat stress applied at the 5th instar. Modification of the key life history traits of both hosts and parasitoids demonstrated a critical role of humidity in modulating the effect of heat stress that has largely been unreported. One possible explanation is that humidity affects host suitability for parasitism through regulating its water budget (Johnson, 2010), indirectly affecting responses in parasitoids. For example, Mainali and Lim (2013) found high (90%–95%) humidity increased the adult emergence of an egg parasitoid *Ooencyrtus nezarae*, due to reduced water loss in host eggs. In our system, *V. canescens* is a koinobiont endoparasitoid, which feeds on host larvae after parasitism, and it is critical for the host to reach the final (5th) instar for the parasitoid to complete its own development and eclose as an adult wasp. The responses of hosts to humidity may further affect their nutritional quality for the development of parasitoids. However, disentangling this effect requires further investigation into how the suitability of hosts for parasitism could be driven by different humidity regimes.

The path analysis (SEM) showed that heat stress affected hosts directly, but indirectly affected the body size of parasitoids through host survival time. Previous studies found that *V. canescens* exhibited flexible growth patterns to accommodate the growth of their hosts (Harvey, 1996; Harvey & Vet, 1997). Therefore, the responses of hosts to heat stress could result in indirect phenotypic responses of parasitoids. To our knowledge, there are few studies examining these direct and indirect effects across two trophic levels. Simaz and Szűcs (2021) found increasing heat wave intensity could directly affect the life history of the host *Halyomorpha halys* and the parasitoid *Trissolcus japonicus*, and indirectly affecting parasitoid emergence in the second generation. However, we demonstrated that the direct and indirect effect of heat stress could be observed even within a single generation, as the host responses are directly related to the ontogenetic development of koinobiont parasitoids (Harvey et al., 1994). Our results showed that humidity may play an important role in moderating the direct and indirect effects of heat stress on hosts and parasitoids, which could have important implications for their co‐existence, extinction risk, population dynamics, and potential for more pest outbreaks under similar conditions in nature.

We attributed the humidity effect to the differences in the average moisture levels between treatments, but variation in humidity, might also affect some critical life history stages such as diapause (Seymour & Jones, 2000; Wetherington et al., 2017). Higher variation in humidity may trigger more humidity‐induced diapause in parasitoids (Wetherington et al., 2017), but in the present study, this effect was not considered. Furthermore, optimum humidity levels, which may depend on the temperature, are unclear in our system. Gross (1988) found a significant decline in parasitoid emergence *Trichogramma pretiosum* over 80% or below 40% RH. Duale (2005) found that the optimum humidity for the development of the parasitoid *Pediobius furvus* was between 60% and 80% RH. There may be a nonlinear effect of humidity on the life history performance of hosts and parasitoids, and so a greater range of humidity by temperature combinations should be explored in the future.

The present study has implications for pest management and biological control. Humid environments may increase the development of hosts and possibly increase the fitness of their adult stage, resulting in more frequent and severe pest outbreaks (Stireman et al., 2005; Wetherington et al., 2017). On the other hand, humid environments may also increase the eclosion of parasitoids, leading to more successful biological control. Experiencing long heat waves, especially at certain larval stages, is detrimental to parasitoids, suggesting that the success of biological control under continuous heat stress events is stage‐specific. However, this effect of heat stress on parasitoids was largely modulated by different humidity regimes. From this point of view, more evaluation of stage‐specific effects of heat events under a multi‐stressor framework may provide practical insights into the successful applications of biological control in the field.

## 5 CONCLUSION

Our findings showed how humidity can play an important role in modifying the life history responses of hosts and parasitoids to heat stress, through a direct effect on the hosts and an indirect effect on parasitoids. Our results highlight the role of humidity in maintaining the success of biological control and importance of accounting for humidity in predicting the population dynamics and distribution of species in the context of climate change and the projected increase in humid heat waves. Our study emphasizes how interactions between environmental stressors can affect trophic interactions through different pathways and in ways that are not predictable from studying single stressors alone, ultimately modifying the interactions between species and their responses to the changing climate. Further disentangling the direct and indirect effects of humidity and heat stress will bring us one step closer to predicting community‐level responses to climate change.

## AUTHOR CONTRIBUTIONS

**Dongbo Li:** Conceptualization (equal); data curation (equal); formal analysis (equal); investigation (equal); methodology (equal); visualization (equal); writing – original draft (equal). **Benjamin Brough:** Investigation (equal). **Jasper W. Rees:** Investigation (equal). **Christophe F. D. Coste:** Conceptualization (equal); writing – review and editing (equal). **Chenggui Yuan:** Conceptualization (equal); writing – review and editing (equal). **Mike S. Fowler:** Conceptualization (equal); funding acquisition (equal); writing – review and editing (equal). **Steven M. Sait:** Conceptualization (equal); funding acquisition (equal); methodology (equal); writing – review and editing (equal).

## CONFLICT OF INTEREST STATEMENT

The authors declare no conflict of interests.

## Supporting information


Appendix S1.

## Data Availability

Data is free to access in Dryad data repository (https://doi.org/10.5061/dryad.0p2ngf28f) and GitHub repository (https://github.com/Dongboli/experimental‐data.git).
