# Supplementary material for: Humidity modifies species‐specific and age‐dependent heat stress effects in an insect host‐parasitoid interaction
Source: Ecol Evol. 2024 Jul 21;14(7):e70047. doi: 10.1002/ece3.70047 (PMC11260500; doi:10.1002/ece3.70047)
Supplement: Supplementary file 1 — Appendix S1. [file ECE3-14-e70047-s001.docx]

**Appendix S1:**

**Humidity modifies age-dependent heat wave effects in an insect host-parasitoid interaction**

**Table of content**

1. **Humidity levels in the incubators**
2. **Supplemental tables for data analysis**
3. **Humidity levels in the incubators**


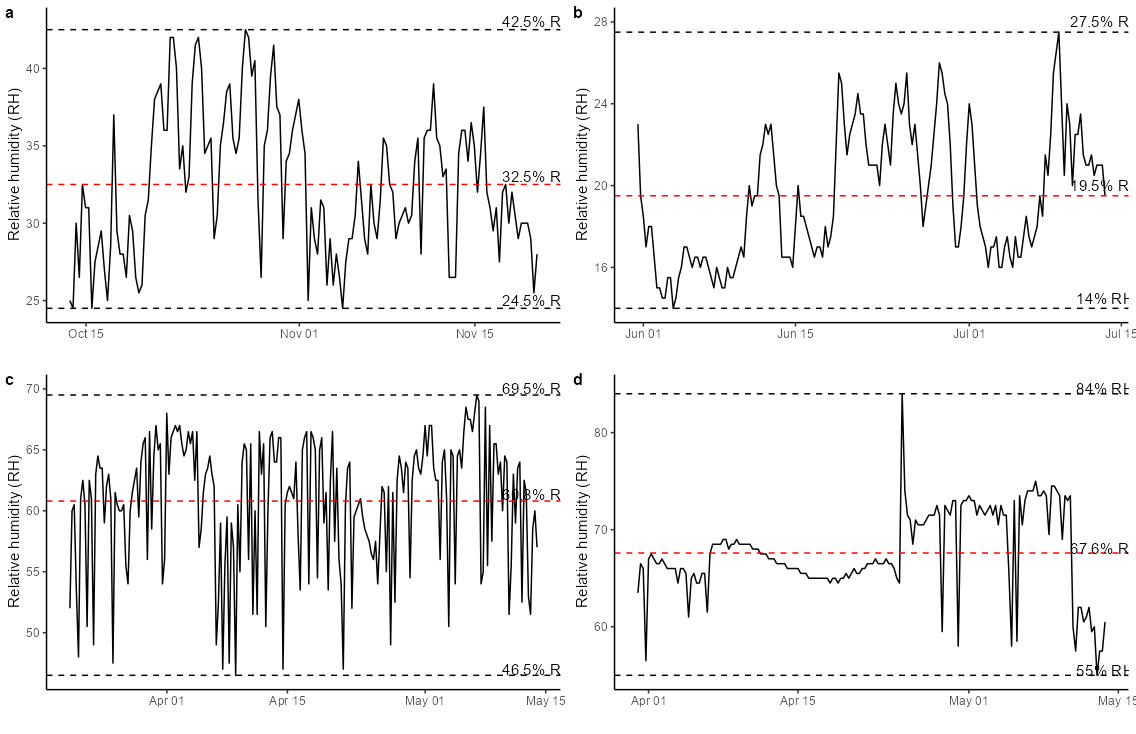


**Figure S1**. Relative humidity (RH) levels change every 6 hours in the incubators with different temperature and humidity manipulation, with max (top dashed line), min (bottom dashed line), and mean (red middle dashed line) RH showed on each panel. ***a***) Constant 28℃ without humidity manipulation: mean = 32.5%, max = 42.5%, min = 24.5%. ***b***) Constant 38℃ without humidity manipulation: mean = 19.5%, max = 27.5%, min = 14%. ***c***) Constant 28℃ with humidity manipulation: mean = 60.8%, max = 69.5%, min = 46.5%. ***d***) Constant 38℃ with humidity manipulation: mean = 67.6%, max = 84%, min = 55%. Note that ***a***) and ***b***) are pilot data which are not corresponding to the period of this experiment.

1. **Supplemental tables for data analysis**

**Table S1** Anova (type III sums of squares) table of the binomial generalized linear model (GLM) on the adult emergence of unparasitized hosts. No significant effects were found.

| Predictors | LR Chisq | Df | *p* |
| --- | --- | --- | --- |
| Humidity | 2.333 | 1 | 0.127 |
| Duration | 1.190 | 1 | 0.275 |
| Larval tage | 1.122 | 1 | 0.290 |
| Humidity: Duration | 0.764 | 1 | 0.382 |
| Humidity: Larval stage | 1.291 | 1 | 0.256 |
| Duration: Larval stage | 0.068 | 1 | 0.794 |
| Humidity: Duration: Larval stage | 0.481 | 1 | 0.488 |

**Table S2** Summary table of the binomial GLM on the adult emergence of unparasitized hosts. No significant effects were found.

| Predictors | Estimate | Std. error | z value | *p* |
| --- | --- | --- | --- | --- |
| Intercept | 4.335 | 1.717 | 2.525 | **0.012** |
| Humidity | -2.415 | 1.869 | -1.292 | 0.196 |
| Duration | -0.456 | 0.463 | -0.984 | 0.325 |
| Larval stage | -1.756 | 1.875 | -0.937 | 0.349 |
| Humidity: Duration | 0.432 | 0.524 | 0.825 | 0.410 |
| Humidity: Larval stage | 2.228 | 2.132 | 1.045 | 0.296 |
| Duration: Larval stage | -0.136 | 0.509 | -0.267 | 0.789 |
| Humidity: Duration: Larval stage | -0.405 | 0.603 | -0.672 | 0.502 |

**Table S3** The estimated marginal linear trends (and 95% Confidence Limits; Lenth 2024) of heat stress duration applied to different larval stages under low- and high-humidity levels, on the changes of probability of host emergence in unparasitized hosts. Trends < 0 indicate decrease in emergence probability for host with increasing heat stress duration (italics); trend estimates formatted in bold showed significant difference from 0 at *p* < 0.05.

| Humdity | Larval Stage | Trend | SE | df | Asymp.LCL | Asymp.UCL |
| --- | --- | --- | --- | --- | --- | --- |
| Low | 4^th^ instar | *-0.458* | 0.463 | Inf | -1.364 | 0.452 |
| High | 4^th^ instar | *-0.024* | 0.244 | Inf | -0.503 | 0.455 |
| Low | 5^th^ instar | ***-0.592*** | 0.211 | Inf | *-1.005* | *-0.179* |
| High | 5^th^ instar | ***-0.565*** | 0.213 | Inf | *-0.982* | *-0.149* |

**Table S4** Summary table of the multinomial logit GLM on the relative success of parasitoids, hosts, and no-emergence in parasitized hosts (‘none’; i.e., both host larvae and developing parasitoid died). Reference level = parasitoid emergence. The multinomial logit coefficients of ‘host adult emergence’ and ‘none’ relative to ‘parasitoid emergence’ were also estimated and compared using two-tailed Wald z-tests (Kwak and Clayton-Matthews, 2002).

| Outcomes | Predictors | Estimates | Std. errors | *z* | *p* |
| --- | --- | --- | --- | --- | --- |
| Host emergence | Intercept | -2.097 | 0.708 | -2.963 | 0.003 |
|  | Humidity | -3.172 | 1.526 | -2.078 | 0.038 |
|  | Duration | 0.962 | 0.259 | 3.713 | 0.002 |
|  | Larval stage (LS) | 0.480 | 0.972 | 0.494 | 0.621 |
|  | Humidity: duration | 0.767 | 0.536 | 1.431 | 0.152 |
|  | Humidity: LS | 3.806 | 1.820 | 2.092 | 0.036 |
|  | Duration: LS | -0.810 | 0.344 | -2.354 | 0.018 |
|  | Humidity: duration: LS | -1.306 | 0.664 | -1.968 | 0.049 |
| None | Intercept | -1.992 | 0.850 | -2.344 | 0.019 |
|  | Humidity | -0.245 | 1.526 | -0.187 | 0.852 |
|  | Duration | 0.395 | 0.329 | 1.967 | 0.231 |
|  | Larval stage (LS) | -1.123 | 1.337 | -0.840 | 0.401 |
|  | Humidity: duration | 0.036 | 0.567 | 0.064 | 0.949 |
|  | Humidity: LS | 1.849 | 1.815 | 1.018 | 0.308 |
|  | Duration: LS | 0.319 | 0.432 | 0.738 | 0.461 |
|  | Humidity: duration: LS | -0.595 | 0.674 | -0.883 | 0.377 |

**Table S5** Pairwise comparisons of the estimated marginal trends (Lenth 2024) for the impact of heat stress duration on host and parasitoid emergence, when heat was applied to either the 4^th^ or 5^th^ instar under different humidity levels. Pairwise differences in the 4^th^ and 5^th^ instar slope estimates, based on a multinomial logit GLM (Table 2, main text), are formatted in bold when they differed significantly from 0.

| Contrast | Outcomes | Humidity | estimate | SE | df | t.ratio | *p* |
| --- | --- | --- | --- | --- | --- | --- | --- |
| 4^th^ instar- 5^th^ instar | Parasitoid | Low | -0.164 | 0.212 | 16 | -0.771 | 0.452 |
| 4^th^ instar- 5^th^ instar | Host | Low | **0.646** | 0.226 | 16 | 2.86 | **0.011** |
| 4^th^ instar- 5^th^ instar | None | Low | -0.483 | 0.272 | 16 | -1.77 | 0.095 |
| 4^th^ instar- 5^th^ instar | Parasitoid | High | **-0.797** | 0.295 | 16 | -2.71 | **0.016** |
| 4^th^ instar- 5^th^ instar | Host | High | **1.32** | 0.361 | 16 | 3.66 | **0.002** |
| 4^th^ instar- 5^th^ instar | None | High | -0.521 | 0.334 | 16 | -1.56 | 0.138 |

**Table S6** Pairwise comparisons of the estimated marginal trends (Lenth 2024) for the impact of heat stress duration on host and parasitoid emergence under low humidity and high humidity levels for different larval stages. Slope estimates come from a multinomial logit GLM (Table 2, main text). No significant differences in slopes between low and high humidity treatments were found.

| Contrast | Outcomes | Larval stage | estimate | SE | df | t.ratio | *p* |
| --- | --- | --- | --- | --- | --- | --- | --- |
| Low- High | Parasitoid | 4^th^ instar | 0.268 | 0.308 | 16 | 0.870 | 0.397 |
| Low- High | Host | 4^th^ instar | -0.499 | 0.330 | 16 | -1.51 | 0.150 |
| Low- High | None | 4^th^ instar | 0.231 | 0.347 | 16 | 0.667 | 0.515 |
| Low- High | Parasitoid | 5^th^ instar | -0.366 | 0.193 | 16 | -1.90 | 0.076 |
| Low- High | Host | 5^th^ instar | 0.173 | 0.269 | 16 | 0.645 | 0.528 |
| Low- High | None | 5^th^ instar | 0.193 | 0.255 | 16 | 0.754 | 0.462 |

**Table S7** Anova (type III sums of squares) table of the gamma GLM with a log-link function on the larval development time of unparasitized hosts and parasitoids. Significant effects were in bold.

|  | Unparasitized host | | | Parasitoid | | |
| --- | --- | --- | --- | --- | --- | --- |
| Predictors | LR Chisq | Df | *p* | LR Chisq | Df | *p* |
| Humidity | 55.966 | 1 | **<0.001** | 1.189 | 1 | 0.275 |
| Duration | 17.101 | 1 | **<0.001** | 4.558 | 1 | **0.033** |
| Larval stage | 0.012 | 1 | 0.912 | 1.943 | 1 | 0.163 |
| Humidity: Duration | 9.067 | 1 | **0.003** | 0.036 | 1 | 0.849 |
| Humidity: Larval stage | 1.713 | 1 | 0.191 | 0.055 | 1 | 0.814 |
| Duration: Larval stage | 3.558 | 1 | 0.059 | 4.794 | 1 | **0.029** |
| Humidity: Duration: Larval stage | 0.184 | 1 | 0.668 | 1.302 | 1 | 0.254 |

**Table S8** Summary table of the gamma GLM with a log-link function on the larval development time of unparasitized hosts and parasitoids. Significant effects were in bold.

|  | Unparasitized host | | | | Parasitoid | | | |
| --- | --- | --- | --- | --- | --- | --- | --- | --- |
| Predictors | Estimate | Std. error | t value | *p* | Estimate | Std. error | t value | *p* |
| Intercept | 3.386 | 0.026 | 128.791 | **<0.001** | 3.045 | 0.024 | 124.655 | **<0.001** |
| Humidity | -0.291 | 0.039 | -7.429 | **<0.001** | -0.049 | 0.045 | -1.094 | 0.276 |
| Duration | 0.036 | 0.009 | 4.110 | **<0.001** | 0.020 | 0.014 | 1.398 | 0.165 |
| Larval stage | -0.004 | 0.037 | -0.111 | 0.912 | -0.068 | 0.032 | -2.123 | **0.036** |
| Humidity: Duration | 0.039 | 0.013 | 2.976 | **0.003** | -0.006 | 0.025 | -0.235 | 0.815 |
| Humidity: Larval stage | 0.071 | 0.055 | 1.301 | 0.195 | -0.010 | 0.054 | -0.189 | 0.850 |
| Duration: Larval stage | -0.024 | 0.013 | -1.877 | 0.062 | 0.035 | 0.016 | 2.177 | **0.032** |
| Humidity: Duration: Larval stage | -0.008 | 0.019 | -0.426 | 0.671 | 0.031 | 0.027 | 1.136 | 0.259 |

**Table S9** The estimated marginal linear trends (and 95% Confidence Limits) of heat stress duration applied to different larval stages under low- and high-humidity levels, on the changes of larval development time in unparasitized hosts and parasitoids. Trends > 0 indicate increase in larval development time for host with increasing heat stress duration; trend estimates formatted in bold showed significant difference from 0 at *p* < 0.05.

|  | Humidity | Larval stage | Duration.trend | SE | Df | Lower.CL | Upper.CL |
| --- | --- | --- | --- | --- | --- | --- | --- |
| Unparasitized host | Low | 4^th^ instar | **0.0361** | 0.00878 | 201 | **0.01878** | **0.0534** |
|  | High | 4^th^ instar | **0.0750** | 0.00968 | 201 | **0.05591** | **0.0941** |
|  | Low | 5^th^ instar | 0.0119 | 0.00946 | 201 | -0.00678 | 0.0305 |
|  | High | 5^th^ instar | **0.0425** | 0.01063 | 201 | **0.02159** | **0.0635** |
| Parasitoid | Low | 4^th^ instar | 0.0204 | 0.01458 | 108 | -0.00852 | 0.0493 |
|  | High | 4^th^ instar | 0.0145 | 0.02050 | 108 | -0.02615 | 0.0551 |
|  | Low | 5^th^ instar | **0.0557** | 0.00715 | 108 | **0.04157** | **0.0699** |
|  | High | 5^th^ instar | **0.0809** | 0.00790 | 108 | **0.06520** | **0.0965** |

**Table S10** Partial pairwise comparisons of the estimated marginal trends (Lenth 2024) for the impact of heat stress duration on larval development time of unparasitized host and parasitoid, when heat was applied to either the 4^th^ or 5^th^ instar under different humidity levels. Pairwise differences in the 4^th^ and 5^th^ instar slope estimates, or under low- and high humidity slope estimates, based on a log-link gaussian GLMs (Table S7-S9), are formatted in bold when they differed significantly from 0.

|  | Contrast | Humidity | estimate | SE | df | t.ratio | *p* |
| --- | --- | --- | --- | --- | --- | --- | --- |
| Unparasitized host | 4^th^ instar- 5^th^ instar | Low | 0.024 | 0.013 | 201 | 1.877 | 0.241 |
|  | 4^th^ instar- 5^th^ instar | High | 0.032 | 0.014 | 201 | 2.257 | 0.112 |
|  | Low- High | 4^th^ instar | -0.039 | 0.013 | 201 | -2.976 | **0.017** |
|  | Low- High | 5^th^ instar | **-**0.031 | 0.014 | 201 | -2.156 | 0.139 |
| Parasitoid | 4^th^ instar- 5^th^ instar | Low | -0.035 | 0.016 | 108 | -2.177 | 0.136 |
|  | 4^th^ instar- 5^th^ instar | High | -0.066 | 0.022 | 108 | -3.022 | **0.016** |
|  | Low- High | 4^th^ instar | 0.006 | 0.025 | 108 | 0.235 | 0.995 |
|  | Low- High | 5^th^ instar | -0.025 | 0.011 | 108 | -2.357 | 0.092 |

**Table S11** Anova (type III sums of squares) table of the gaussian GLM with a log-link function on the body size of unparasitized hosts and parasitoids. Significant effects were in bold.

|  | Unparasitized host | | | Parasitoid | | |
| --- | --- | --- | --- | --- | --- | --- |
| Predictors | LR Chisq | Df | *p* | LR Chisq | Df | *p* |
| Humidity | 26.878 | 1 | **<0.001** | 8.773 | 1 | **0.003** |
| Duration | 0.445 | 1 | 0.505 | 2.120 | 1 | 0.145 |
| Larval stage | 4.913 | 1 | **0.027** | 5.148 | 1 | **0.023** |
| Humidity: Duration | 15.586 | 1 | **<0.001** | 1.851 | 1 | 0.174 |
| Humidity: Larval stage | 8.336 | 1 | **0.004** | 2.984 | 1 | 0.084 |
| Duration: Larval stage | 0.751 | 1 | 0.386 | 6.856 | 1 | **0.009** |
| Humidity: Duration: Larval stage | 5.566 | 1 | **0.018** | 2.219 | 1 | 0.136 |

**Table S12** Summary table of the gaussian GLM with a log-link function on the body size of unparasitized hosts and parasitoids. Significant effects were in bold.

|  | Unparasitized host | | | | Parasitoid | | | |
| --- | --- | --- | --- | --- | --- | --- | --- | --- |
| Predictors | Estimate | Std. error | t value | *p* | Estimate | Std. error | t value | *p* |
| Intercept | 0.506 | 0.028 | 18.174 | **<0.001** | 0.452 | 0.029 | 15.742 | **<0.001** |
| Humidity | 0.189 | 0.037 | 5.100 | **<0.001** | 0.144 | 0.048 | 3.032 | **0.003** |
| Duration | 0.006 | 0.009 | 0.660 | 0.510 | 0.024 | 0.017 | 1.439 | 0.153 |
| Larval stage | 0.081 | 0.037 | 2.206 | **0.029** | 0.083 | 0.037 | 2.246 | **0.027** |
| Humidity: Duration | -0.050 | 0.013 | -3.901 | **<0.001** | -0.037 | 0.027 | -1.370 | 0.173 |
| Humidity: Larval stage | -0.142 | 0.049 | -2.871 | **0.005** | -0.100 | 0.058 | -1.738 | 0.085 |
| Duration: Larval stage | -0.011 | 0.013 | -0.865 | 0.388 | -0.048 | 0.019 | -2.577 | **0.011** |
| Humidity: Duration: Larval stage | 0.042 | 0.018 | 2.346 | **0.020** | 0.044 | 0.029 | 1.497 | 0.137 |

**Table S13** The estimated marginal linear trends (and 95% Confidence Limits) of heat stress duration applied to different larval stages under low- and high-humidity levels, on the changes of body size in unparasitized hosts and parasitoids. Trend < 0: decrease in body size of unparasitized host or parasitoid with increasing heat stress duration (italic); Trend > 0: increase in body size of unparasitized host or parasitoid with increasing heat stress duration; trend estimates formatted in bold showed significant difference from 0 at *p* < 0.05.

|  | Humidity | Larval stage | Duration.trend | SE | Df | Lower.CL | Upper.CL |
| --- | --- | --- | --- | --- | --- | --- | --- |
| Unparasitized host | Low | 4^th^ instar | 0.0063 | 0.0095 | 157 | -0.0125 | 0.0250 |
|  | High | 4^th^ instar | ***-0.0441*** | 0.0088 | 157 | **-0.0614** | **-0.0268** |
|  | Low | 5^th^ instar | *-0.0048* | 0.0086 | 157 | -0.0219 | 0.0122 |
|  | High | 5^th^ instar | *-0.0131* | 0.0090 | 157 | -0.0309 | 0.0047 |
| Parasitoid | Low | 4^th^ instar | 0.0244 | 0.0170 | 107 | -0.0092 | 5.80e-02 |
|  | High | 4^th^ instar | *-0.0126* | 0.0210 | 107 | -0.0542 | 2.90e-02 |
|  | Low | 5^th^ instar | ***-0.0241*** | 0.0081 | 107 | **-0.0403** | **-7.91e-03** |
|  | High | 5^th^ instar | *-0.0169* | 0.0086 | 107 | -0.0339 | 1.48e-05 |

**Table S14** Partial pairwise comparisons of the estimated marginal trends (Lenth 2024) for the impact of heat stress duration on the body size of unparasitized host (i.e., mid femur) and parasitoid (i.e., hind tibia), when heat was applied to either the 4^th^ or 5^th^ instar under different humidity levels. Pairwise differences in the 4^th^ and 5^th^ instar slope estimates, or under low- and high humidity slope estimates, based on a log-link gaussian GLMs (Table S7-S9), are formatted in bold when they differed significantly from 0.

|  | Contrast | Humidity | estimate | SE | df | t.ratio | *p* |
| --- | --- | --- | --- | --- | --- | --- | --- |
| Unparasitized host | 4^th^ instar- 5^th^ instar | Low | 0.011 | 0.013 | 157 | 0.865 | 0.823 |
|  | 4^th^ instar- 5^th^ instar | High | -0.031 | 0.013 | 157 | -2.468 | 0.069 |
|  | Low- High | 4^th^ instar | 0.050 | 0.013 | 157 | 3.901 | **0.001** |
|  | Low- High | 5^th^ instar | 0.008 | 0.013 | 157 | 0.663 | 0.911 |
| Parasitoid | 4^th^ instar- 5^th^ instar | Low | 0.048 | 0.019 | 107 | 2.577 | 0.054 |
|  | 4^th^ instar- 5^th^ instar | High | 0.004 | 0.023 | 107 | 0.192 | 0.931 |
|  | Low- High | 4^th^ instar | 0.037 | 0.027 | 107 | 1.370 | 0.521 |
|  | Low- High | 5^th^ instar | -0.007 | 0.012 | 107 | -0.604 | 0.931 |

**Table S15** Anova table of group-wide interaction paths with humidity in piecewise SEM. Significant values indicated models were unconstrained (i.e., paths were different with different humidity levels) and unsignificant values indicated models were constrained to the global model (i.e., paths were not different with different humidity levels). Note that the global goodness-of-fit were unavailable due to 0 degree of freedom.

| Response | Predictor | Test.Stat | DF | *p* value |
| --- | --- | --- | --- | --- |
| Host_larval | Duration: Humidity | 229.7 | 1 | 0.0005 |
| Host_larval | Humidity: Stage | 229.7 | 1 | 0.0453 |
| Wasp_size | Host_larval:Humidity | 9.4 | 1 | 0.5063 |
| Wasp_size | Humidity:Duration | 9.4 | 1 | 0.1627 |
| Wasp_size | Humidity:Stage | 9.4 | 1 | 0.3566 |

**Table S16** Estimated coefficients from multigroup piecewise SEM. Standard estimate (scaled standardization) are only available for path ‘Duration – Wasp_size’ and ‘Host_larval – Wasp_size’. c = constrained

| **Group [high humidity] coefficients:** | | | | | | | |
| --- | --- | --- | --- | --- | --- | --- | --- |
| Response | Predictor | Estimate | Std.Error | DF | Crit. value | *p* value | Std. Estimate |
| Host_larval | Duration | 0.0026 | 2e-04 | 57 | 12.4189 | 0.0000 | - |
| Host_larval | Stage | - | - | 1 | 0.8858 | 0.3466 | - |
| Host_larval | Stage = 4^th^ instar | 3.4811 | 0.0096 | 57 | 361.9006 | 0.0000 | - |
| Host_larval | Stage = 5^th^ instar | 3.493 | 0.0073 | 57 | 476.7709 | 0.0000 | - |
| Wasp_size | Host_larval | -0.0152 | 0.0025 | 111 | -6.1729 | 0.0000 | -0.3429 c |
| Wasp_size | Duration | 2e-04 | 3e-04 | 111 | 0.4663 | 0.6419 | 0.042 c |
| Wasp_size | Stage | - | - | 1 | 0.0754 | 0.7836 | - c |
| Wasp_size | Stage = 5^th^ instar | 0.5467 | 0.5467 | 56 | 50.9409 | 0.0000 | - |
| Wasp_size | Stage = 4^th^ instar | 0.5596 | 0.5596 | 56 | 40. 8614 | 0.0000 | - |

| **Group [low humidity] coefficients:** | | | | | | | |
| --- | --- | --- | --- | --- | --- | --- | --- |
| Response | Predictor | Estimate | Std.Error | DF | Crit. value | *p* value | Std. Estimate |
| Host_larval | Duration | 0.0017 | 2e-04 | 52 | 9.4774 | 0.0000 | - |
| Host_larval | Stage | - | - | 1 | 4.0784 | 0.0432 | - |
| Host_larval | Stage = 5^th^ instar | 3.6074 | 0.0066 | 52 | 543.9108 | 0.0000 | - |
| Host_larval | Stage = 4^th^ instar | 3.6278 | 0.0067 | 52 | 545.4897 | 0.0000 | - |
| Wasp_size | Host_larval | -0.0152 | 0.0025 | 111 | -6.1729 | 0.0000 | -0.2336 c |
| Wasp_size | Duration | 2e-04 | 3e-04 | 111 | 0.4663 | 0.6419 | 0.0428 c |
| Wasp_size | Stage | - | - | 1 | 0.0754 | 0.7836 | - c |
| Wasp_size | Stage = 4^th^ instar | 0.4751 | 0.0156 | 51 | 30.4918 | 0.0000 | - |
| Wasp_size | Stage = 5^th^ instar | 0.4892 | 0.0151 | 51 | 32.4198 | 0.0000 | - |

**References**

KWAK, C. & CLAYTON-MATTHEWS, A. 2002. Multinomial logistic regression. *Nursing research*, 51, 404-410.

LENTH, R.V. (2024). *emmeans: Estimated Marginal Means, aka Least-Squares*

*Means*. R package version 1.10.2, https://CRAN.R-project.org/package=emmeans
